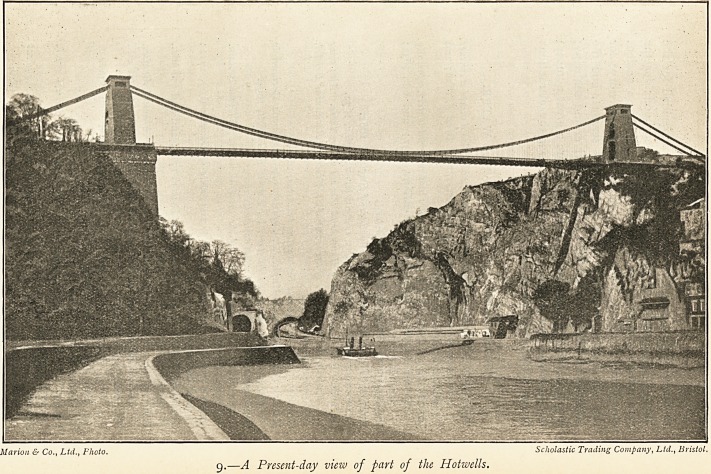# The Reputation of the Hotwells (Bristol) as a Health-Resort

**Published:** 1902-09

**Authors:** L. M. Griffiths


8.—*Bristol Hotwells in 1822.*

### The Bristol Medico-Chirurgical Journal.

"*Scire est nescire, nisi id me*
*Scire alius sciret.*"

SEPTEMBER, 1902.

### THE REPUTATION OF THE HOTWELLS (BRISTOL) AS A HEALTH-RESORT.

BY

L. M. Griffiths, M.R.C.S. Eng.

(*Continued from p. 152.*)

In 1798, after "much difficulty in finding a home for it, on account of a popular impression that 'medicated airs' were explosive, and that the complaints, which were to be cured by inhaling them, might be infectious,"[1] the contemplated Pneumatic Institution of Beddoes was opened in Dowry Square.[2]

[1] *Thomas Poole and his Friends.* By Mrs. Henry Sandford, 1888, vol. i., pp. 253-4.

[2] "The house in the corner, forming the north angle of the Square" (Cottle's *Early Recollections*, 1837, ii. 29). The following announcement is in the *Bristol Directory* for 1799–1800:—

Hotwells
Medical Pneumatic Institution.

*Physicians*—T. Beddoes, M.D. R. Kinglake, M.D. P. Roget, M.D.
*Medical Superintendant*—H. Davy. *Attendant*—P. Dwyer.

Roget, who was then only twenty years of age, afterwards became famous as the compiler of the well-known *Thesaurus of English Words and*

The necessary funds had been completed by a gift of £1,000 from Thomas Wedgwood, who was a patient of Beddoes and was then living at Cote House with his brother John. He said that it would be worth that sum to be sure "that the elastic fluids would *not* be serviceable in medicine."[1] Humphrey Davy,[2] then only nineteen years of age, was its superintendent.

*Phrases*; he lived on or near the premises (*Bristol Directory*, 1799-1800, p. 6). In 1800 out-patients were received every Sunday and Wednesday morning (*The Medical and Physical Journal*, 1800, iii. 488), and accommodation was provided for two or three paralytic in-patients, as the inhalation of nitrous oxide "was peculiarly serviceable to persons labouring under palsy" (*Anti-Jacobin Review and Magazine*, 1800, vi. 425). In the 1805 Directory the word "Pneumatic" did not appear in the title. Beddoes and J. E. Stock, the future biographer of Beddoes, were then physicians, and John King was surgeon. The days of attendance were Sundays at Dowry Square, and Mondays, Wednesdays, and Fridays at Little Tower Court, Broad Quay. In 1806 there is no mention of any attendance at the Hotwells. In 1809, the year after the death of Beddoes, its title is "Preventive Medical Institution," and Broad Quay only is given as the place of the Institution. In the 1813 Directory it is not mentioned.

1 Mrs. Henry Sandford's *Thomas Poole and his Friends*, vol. i., p. 254. Thomas Wedgwood and his other brother Josiah became responsible for an annuity of £150 to Coleridge, the trustee for which was the Rev. John Prior Estlin, Unitarian minister at Lewin's Mead, and master of a successful school on St. Michael's Hill. Mr. Estlin's family had a marked influence on the medical life of the city. Dr. James Cowles Prichard married one of his daughters. His son, John Bishop Estlin, who became famous as an oculist, was the founder of the Bristol Eye Dispensary.

2 Davy came to Clifton in October, 1798, and stayed with Beddoes. At the beginning of 1799 he went to live at the Pneumatic Institution (Dr. T. E. Thorpe's *Humphrey Davy*, 1896, p. 40). It was here that he made his famous investigations into the properties of nitrous oxide, which had been discovered by Priestley in 1774. In 1799 Maria Edgeworth, a sister of whom Beddoes had married, was staying in Clifton with Mrs. Edgeworth, who, in reference to the experiments, wrote: "A young man, a Mr. Davy, at Dr. Beddoes', who has applied himself much to chemistry, has made some discoveries of importance, and enthusiastically expects wonders will be performed by the use of certain gases, which inebriate in the most delightful manner, having the oblivious effects of Lethe, and at the same time giving rapturous sensations of the Nectar of the Gods!" (*Life and Letters of Maria Edgeworth*, 1894, vol. i., pp. 65–6). And on July 12, 1799, Southey, writing to his brother an excited account of Davy's results, said: "Davy has actually invented a new pleasure, for which language has no name" (*Life and Correspondence of Robert Southey*, 1850, vol. ii., p. 21). Although its capabilities for surgical anæsthesia were recognised, it was, as its popular name implies, mostly used as an amusement. Its application was made the subject of a caricature by Gillray in 1802.

The treatment was not entirely, or even mainly, pneumatic. That was reserved only for "those patients who seemed to derive no benefit from the administration of medicines in the common form."[1] In 1799 Beddoes issued a pamphlet, *Notice of Some Observations made at the Medical Pneumatic Institution*; in this, with his characteristic energy, he defended[2] his plans, notwithstanding the failures that had occurred. That he himself soon lost faith in his special treatment by inhalation may be gathered from his acknowledgment "that the trials that have been made of factitious airs and vapours, seem, as yet, very far from having produced any thing like a successful mode of treating consumption,"[3] and Dr. Stock, in his biography of Beddoes, says that in 1803 "the pneumatic practice was almost abandoned,"[4] and the institution became an ordinary dispensary,[5] with the special feature of practical instruction in the art of preserving health.[6]

[1] *Memoirs of the Life of Thomas Beddoes, M.D.* By John Edmonds Stock, M.D., 1811, p. 157.

[2] *Op. cit.*, p. 177.

[3] *Essay on the Causes, Early Signs, and Prevention of Pulmonary Consumption*, 1799, p. 264.

[4] *Op. cit.*, p. 302.

[5] "In order that the trials might be deliberately proceeded in, a fortunate thought occurred to Dr. Beddoes: namely, not to bribe, but to reward all persevering patients; for before the Pneumatic institution was broken up, they allowed every patient sixpence per diem" (Cottle's *Early Recollections*, 1837, ii. 41). Although Cottle states that he learned this from Davy, Miss Meteyard says (*A Group of Englishmen*, 1871, p. 91) it was not exactly the case, and that the money the patients received was the return, under certain conditions, of the half-crown which had to be deposited upon entrance. As an indication of the range of observation at the institution, and of the thoroughness of his work, may be noticed the "Plan of a Public Scrutiny of certain Medicines, lately proposed as anti-venereal, at the Pneumatic Institution near Bristol," which Beddoes put forth in July, 1801 (*The Medical and Physical Journal*, 1801, vi. 165). Vaccination was also done there, and Beddoes, who hesitated at first to adopt it, advocated a national subscription for Jenner, and he said that "probably those very Members of Parliament who from a sense of duty, shewed themselves most sparing of the public purse, will be among the most forward to open their own" (*Ibid*, 1802, viii. 7). The 122 cases during December, 1801, are specified in *The Medical and Physical Journal*, 1802, vii. 301–2, and a classified list of the cases of the 678 patients who attended from January 1st to April 18th, 1802, is given in *Hygëia*, vol. ii., 1808; Essay vi., p. 96.

[6] On March 3rd, 1800, Beddoes issued a circular announcing a series of "Lectures on the Laws of Animal Nature, and of the Means of preserving

As a remedy in phthisis Beddoes had no faith in the Hotwell water. In his *Essay on Consumption* he says: "As to the efficacy of this or that spring, in any period of consumption, there is nothing in the pagan and popish legends concerning consecrated fountains and holy water, more absurd than such a persuasion." [1] But accepting the views of Dr. Nathan Drake [2] and of Dr. Richard Fowler [3] that digitalis, "by its almost uniform effect in rendering the action of the arteries more slow than natural, at the same time that it seems to excite the absorbents," [4] and believing that many cases of phthisis had been considerably relieved by its administration, he hoped that "consumption will henceforward as regularly be cured by the fox-glove, as ague by peruvian bark." [5]

Fashions change. But rich and educated persons are ever ready to place themselves under extraordinary and disagreeable conditions in deference to the medical theories of the time. Although Beddoes had practically given up his treatment by factitious airs, he was still strongly of opinion that air modified in some form was essential for the successful treatment of phthisis, and it is interesting to note some of the ways in which the System from Injury upon the most Important Occasions of Life' (*The Medical and Physical Journal*, 1800, iii. 487). In 1803 he put forth "The Rules of the extended Medical Institution for the Benefit of the Sick and Drooping Poor, with an Explanation of its peculiar Design, and various necessary Instructions." (For much interesting information concerning this see *The Medical and Physical Journal*, 1803, x. 571–3, and Stock's *Life of Beddoes*, pp. 318–30.)

1 *Loc. cit.*

2 Drake was a man of literary tastes. His *Shakespeare and his Times* is a well-known and much-valued book.

3 *Contributions to Physical and Medical Knowledge, principally from the West of England*, 1799, pp. 473–520. The profits of this work for the first two years were to be for the benefit of the Pneumatic Institution, and afterwards to "be given to some infirmary within the district" (*Annals of Medicine*, *1798*, iii. 464). It was printed by Biggs and Cottle, who also printed other work of Beddoes. Joseph Cottle, who was in business at the corner of High Street and Corn Street, and who so substantially aided Coleridge, Southey, and Wordsworth, was himself a poet of considerable merit. Many of the passages in his *Alfred* are good. Byron made a virulent and an unjustifiable attack upon him in *English Bards and Scotch Reviewers*, but calls him Amos, which was the name of his brother, who also wrote verse. The same mistake is made in *The Anti-Jacobin*. (See Pryce's *History of Bristol*, 1861, p. 547.)

4 *Essay on Consumption*, 1799, p. 269. 5 P. 270.

this idea was carried out in Clifton under his supervision. Having read that "a lady, in the last stage of consumption, had her distressing symptoms all removed, from living the winter in a room with four cows," and knowing that "in many countries *cow's breath* is a traditionary remedy," Beddoes in 1796 [1] was preparing to give his patients the advantages to be obtained from these proceedings. Starting from the prevailing persuasion that a "residence in hotter countries is beneficial to British invalids," he convinced himself that the cow-house would provide "an atmosphere permanently modified, of a regular temperature," supplied by "the gasses, given out by the fermenting mass of vegetable and animal substances." [2] Obviously the proceeding involved difficulties. "A gentleman in the last stage of consumption, mortified by the refusal of the master of a lodging house to admit cows into it, quitted Clifton." [3] The case of Mrs. Finch, daughter of Dr. Joseph Priestley, is recorded at great length. In 1799 "a stable adjoining to one of the houses in Gloucester Row, twenty feet long, fourteen wide, and nine high, with a small recess, was engaged; and a space sufficient to contain a moderate bed, with a little room to place a table and move about, was partitioned off; and this part was raised, by coarse boards, a few inches above the ground of the stable. Two cows were first placed in the other part of the building, for a few days before Mrs. Finch took up her abode in it" [4] about the end of September. Upon her entry her case was looked upon as hopeless. On October 8th she wrote that it was "a much more comfortable abode than she had formed an idea of. So different have been her feelings from those of the last six months, that she should reluctantly change her apartment for the *night*, however she might wish a *cleaner* and more *chearful* one for the *day*." [5] She lived there for about six months, with the exception of a few days. In the summer of 1800 she felt perfectly well. During the following winter she "confined herself to an apartment,

[1] *Considerations on Factitious Airs*, Part IV., 1796, pp. 121–2.

[2] *Observations on the Medical and Domestic Management of the Consumptive*, 1801, pp. 21, 22, 68.

[3] P. 24. [4] P. 46. [5] P. 49.

artificially heated, and continued to enjoy entire freedom from pulmonary complaints till March, 1801, when she got a violent catarrh." She said that till then "I had passed the winter with great credit to the cow-house, the *air* of which I still prefer to my warm room, though it is of a good size, and lies to the sun."[1] Several other cases are recorded of patients living with cows. The results varied, and Beddoes recommended a modification of some of the conditions. In some instances he excluded the cows, and placed the patient in a stable with two stalls, one of which "was filled very full of the materials used by gardeners for hot-beds; in the other the patient's bed was placed."[2] And later he said that "vessels containing the fermentable substances could easily be introduced into a warm apartment; the former as easily be regulated by covers, and the vessels removed entirely, the moment the exhalations appeared to disagree."[3]

That modern experimenters in the treatment of phthisis have no monopoly of eccentricity in this particular direction is amply shown by the practice of Beddoes. They have his zeal, and, using almost his very words, they are determined, as he was, to fight disease with any weapons that seem to offer the least chance of victory. And like him they are living up to the psychological attitude of the moment. It will be interesting to know what the chronicler of the year 2002 will say about them. Future investigators will find in the work of their predecessors plenty of warning and of example; but as the prophylaxis and treatment of phthisis have not yet nearly

[3] Pp. 86-7. The exertions of Beddoes in this direction drew forth an ardent eulogy from a writer in *The Medical and Physical Journal* (1802, vii. 8), who praised him for his efforts in protesting against "the unavailing slavery of routine practice, and for having taught that the province of medicine must be a great and generous science, never a contending trade." Southey, writing in 1799, said: "The faculty dislike Beddoes, because he is more able, and more successful, and more celebrated, than themselves, and because he labours to reconcile the art of healing with common sense, instead of all the parade of mystery with which it is usually enveloped. Beddoes is a candid man, trusting more to facts than reasonings: I understand him when he talks to me, and, in case of illness, should rather trust myself to his experiments than be killed off *secundem artem*, and in the ordinary course of practice" (*Life and Correspondence of Robert Southey*, 1850, vol. ii., pp. 23–4).

reached finality, coming generations should be grateful for all previous independent observations. The world is indebted to every honest and sincere worker, even if a great part of the work has to be superseded.

> "The heights by great men reached and kept
> Were not attained by sudden flight,
> But they, while their companions slept,
> Were toiling upward in the night.
>
> * * * *
>
> Nor deem the irrevocable Past
> As wholly wasted, wholly vain,
> If, rising on its wrecks, at last
> To something nobler we attain."[1]

Dangers to be avoided in each age are generalising on insufficient data and thinking that the last word is being said. Scientific knowledge seems to be no guarantee against erratic developments; for not only are the observers of to-day in the front rank, but Beddoes also was a man of exceptional attainments, and he "spared neither his faculties nor his credit in behalf of the sick." His writings, enforced with much eloquence, combined with satire and also the saving grace of humour, show that he was a man of rare insight into human nature, with its marvellous alternations of health and disease,[2] but he had in a marked degree the defects of his qualities. His enthusiasm[3] in his work often led him to attribute virtues to

[1] Longfellow, "The Ladder of St. Augustine.'

[2] The application of external cold in the treatment of disease had his earnest support. "The public might advantageously forego a considerable proportion of the rare exotic articles in any existing pharmacopœia for the sake of so vulgar a domestic production as ice. When men are better instructed in the laws of their own nature, they will be less eager about ice as a luxury than as a powerful instrument of health. Public ice-houses will be constructed in our cities, towns, and villages" (*Observations on the Medical and Domestic Management of the Consumptive*, 1801, pp. 16–7). He considered it "practicable to acquire measures of irritability and sensibility," and had an instrument "so constructed, as when applied to some artery, to shew the force of its stroke" (*Op. cit.*, p. 197).

[3] Attention should be given to his collective investigation on Influenza, the record of which covers about 130 pages of *The Medical and Physical Journal*, 1803; but his promised Commentary on the returns, which numbered over 120 from various districts, did not appear. There had been a good deal of influenza in Bristol in the early part of 1803, an account of which Nott had published (see *Ibid*, 1803, x. 80–2).

remedies which further observation showed they did not possess. Beddoes was busy with his investigations and with his pen up to the time of his death, which took place in 1808, at 3 Rodney Place, where he had been in practice for some years. He was only 48.[1]

The literary requirements of the fashionable throng that frequented the Hotwells were not great, but they were not altogether overlooked. The 5s. subscription which enabled the visitor to walk in the rooms and gardens also included the privilege of reading the newspapers which were supplied. In addition there was a circulating library kept by Mrs. Ann Yearsley, who, born about 1756, was in early life a milkwoman, but who left that useful but humble occupation and became a minor poet. Some of her verse attracted the favourable notice of Hannah More, by whose aid she published a volume of poems, and with the proceeds of this she started the library. Other books, including a play and a novel, followed later. A portrait which is in the possession of the Bristol Museum and Library shows Mrs. Ann Yearsley as the possessor of considerable personal attractions. She died in 1806.

If it is true that Jack Cade considered that the man who had erected a grammar school had most traitorously corrupted the youth of the realm, that any one who caused printing to be used or a paper mill to be built had committed a grave offence, and that those were most worthy to live who could not read, he had a like-minded successor in one who would have disclaimed any relationship to him. For the same spirit breathes in Sir Anthony Absolute, who declared that "a circulating library is as an evergreen tree of diabolical knowledge." If there is any truth in this dictum, the dwellers in Clifton about this time must have been in a parlous state, for there were at least two institutions of the kind. The clients of these could also make their purchases from "an extensive assortment of

[1] Those who wish to see an impartial and candid survey of the life and work of Beddoes should read the obituary notice which appeared in *The Medical and Physical Journal* (1809, xxi. 183-92). Coleridge, referring to the death of Beddoes, says in a letter to Thomas Poole, Feb. 3, 1809: "Poor Beddoes! he was good and beneficent to all men, but to me he was, moreover, affectionate and loving" (*Letters of Samuel Taylor Coleridge*, 1895, vol. ii., p. 544).

foreign and English perfumery, jewellery, hardwares, toys and stationery."

The Bristol Library Society, which had been formed in 1772 on subscription lines and which was housed in the City Library[1] in King Street, had among its members at the end of the century Southey and Coleridge and Humphrey Davy. The Registers which are still in existence give the names of books taken out by them.[2]

But the glories of the Hotwells were now about to vanish. Soon after 1790, when new arrangements were made between the Merchants' Society and the tenant, necessitating on the latter's part a considerable charge for drinking the water, the pleasure-seekers departed to other places, and the Hotwells was the resort of invalids only, and although the praises of the site were sounded in 1800 by Dr. William Saunders in his book on mineral waters,[3] and endorsed by Dr. William Nisbet in 1804 in *A Medical Guide for the Invalid*, it was all of no avail. Saunders pointed out that "Bristol water, besides being employed medicinally at the spring head, which is in fact but a small part of its consumption, is used largely at the table at the Hotwells, and for all domestic purposes. Its softness, or freedom from earthy salts, is almost proverbially known; and from its excellent quality of keeping untainted for a great length of time in hot climates, it forms a most valuable water for long voyages, and is accordingly exported in great quantities to distant parts."[4] He showed that supposing half a pint of the water to be taken four times in the day, "the patient will have

[1] Only men could use the library. Maria Edgeworth (*op. cit.*, vol. i., p. 18), writing on December 29th, 1791, says that her "father has got a *transfer* of a ticket for the Bristol library, which is an extremely fine one; and what makes it appear ten times finer is, that it is very difficult for strangers to get into. From thence he can get almost any book for us he pleases, except a few of the most scarce, which are by the laws of the library immovable. No ladies go to the library, but Mr. Johns, the librarian, is very civil, and my mother went to his rooms and saw the beautiful prints in Boydell's Shakespear."

[2] See *Chambers's Journal*, 1 Feb., 1890, and *The Library*, vol. v., 1893.

[3] *A Treatise on the Chemical History and Medical Powers of some of the most celebrated Mineral Waters.*

[4] Second Edition, 1805, p. 115.

added to his daily ingesta about five grains and a half of purgative salts; six grains and a half of calcareous salts; and about a quarter of a pint in bulk of carbonic acid; the whole dissolved in a quart of water of the temperature of 74°."[1] By Saunders's time a view somewhat saner than that of most of its earlier eulogists[2] was being taken. He says: "It is not easy to determine how much may be owing to the favourable situation and mild temperate climate which Bristol enjoys; but it cannot be doubted that the Hotwell water, though by no means a cure for consumption, alleviates some of the most harassing symptoms in this formidable disease. We are not yet fully acquainted with the medical virtues which we may expect from the union of a small quantity of carbonic acid with water; but from comparing the effects resulting from this gaseous acid when in a larger dose, and giving very sensible properties to the water with which it is combined, there appears to be some reason for attributing to this substance, a part at least of the virtues of Bristol water."[3] Nisbet endorses the views of his "respectable friend, Dr. Saunders," and corroborates his opinion that "in those affections of the alimentary canal which arise from a residence in a warm climate, whether attended with bilious symptoms or not, the waters are eminently useful. The same advantage they display in diarrhœa and slight attacks of dysentery. In diabetes, if not curing, they are at least a serviceable palliative, and give effect to the powers of other remedies. In consumption, there are some authenticated cases of cure where the disease was in its commencement, and the constitution was [not] broken down; but where it has made progress, and hectic symptoms are far advanced, little is to be expected from this mineral, or any other remedy. But even in these deplorable circumstances it will have some palliative influence over the hectic symptoms, and tend to allay the thirst, feverish heat, and other symptoms of increased temperature."[4] In words taken direct from "*Phillips's Guide*,"[5] Nisbet recom-

[1] Pp. 122-3.

[2] Fothergill was notably one whose views were fairly rational.

[3] Pp. 124-5. [4] P. 46.

[5] *A Guide to all the Watering and Sea-Bathing Places.* By the Editor of the Picture of London. Printed for Richard Phillips. [1803.]

mends invalids of every description to resort to Clifton, which is said to be a "beautiful village, which for the purity and salubrity of its air, has been denominated the Montpelier of England, from its elevated situation furnishes the most charming views over the western part of Bristol, and of the Avon for a considerable way, with its moving scene of ships. It stands on a hill, which rises by a gradual ascent from the river, and is, in a great measure, covered with villas, and elegant piles of buildings. The principal situations for those invalids who prefer this airy abode, are Sun [? Sion] -row and Gloucester-place, on Clifton Down; the Prince of Wales's Crescent; The Mall, which may be regarded as the principal beauty of Clifton; Rodney-place; Boyce's-buildings; York-buildings, etc., etc."[1]

In 1816, according to Carrick, "many houses, and even whole rows, are unoccupied." Notwithstanding the strong protests of Carrick and others against the evil policy of sending patients in advanced phthisis to the Hotwells, the practice of doing so became more common, with of course as a result, a large death-rate, which gradually made the place unpopular. The condition of things is thus pictured by Carrick:—"From the day that the Hotwell became practically a fountain sealed to the lips of every one but the actually moribund, the fame of the place began rapidly to decline. None who drank of the Lethean waters were thenceforth found to recover; because none did drink of them but such as were past recovery. It was now one uniform black list of disappointment and death; and in the course of a very few years it became all over the kingdom a source of horror and despair, instead of hope and confidence, to be ordered to the Hotwells, from whose awful bourne no traveller now returned." [2]

In spite of better facilities of approach and the building of an improved Pump-Room,[3] with the provision of baths, the place became practically deserted. The decay of the Hotwells as a

[1] P. 264.

[2] Extract from letter by Carrick to the Merchants' Society in 1816, "first published in the *Bristol Times* of October 18, 1862."—Mr. Latimer's *The Annals of Bristol in the Nineteenth Century*, 1887, p. 72.

[3] River improvements necessitated the demolition of the New Pump-Room in 1867.

fashionable resort seems to date from the Merchants' Society's new lease in 1790, and the exorbitant charges which were then the rule for all visitors' requirements; but its end no doubt was hastened, as Mr. Latimer points out, by the quieter condition of European politics which enabled persons to visit the continental spas in safety.

In this *Journal* for June, 1889, Dr. John Beddoe summarises the more recent views concerning the Hotwell water, in which "the chief constituent, the carbonate of lime, preponderates over the sulphate," thus varying the relation of those ingredients in the Bath water. Dr. Beddoe quotes Herapath's analysis of the Bristol water, which gave per gallon:

| | |
|---|---|
| "Carbonate of lime ... ... ... ... | 17.7 grains |
| Sulphate of lime ... ... ... ... ... | 9.87 ,, |
| Chloride of sodium ... ... ... ... | 5.89 ,, |
| Sulphate of soda ... ... ... ... | 3.01 ,, |
| Chloride of magnesium ... ... ... | 2.18 ,, |
| Nitrate of magnesia ... ... ... ... | 2.91 ,, |

with smaller quantities of carbonate of magnesia, and of iron, of bitumen, and of silica, and some impregnation of carbonic acid and of nitrogen gas. Mr. F. W. Stoddart found the same aggregate amount of solids as Herapath did, but with slight differences in proportion—rather more sulphuric acid, and less of nitric acid and chlorine; rather less lime, and more magnesia and silica." Owing to the large amount of quarrying that has been done at St. Vincent's Rocks ruining the place at which the water originally came forth, much doubt has been expressed about the site of the spring which attracted to the Hotwells its fashionable crowds; but Dr. Beddoe considers that the well has been recovered, and it is much to be desired that its old popularity should be revived.

A vigorous and well-meant effort to restore the glories connected with the Hotwell spring has within the last few years been made by the erection of the Clifton Spa, an institution replete with modern luxury, and thoroughly equipped with an elaborate system of baths. But even if persons do not require medical treatment, many of them might, for their holiday resort or permanent residence, do well

Marion & Co., Ltd., Photo. Scholastic Trading Company, Ltd., Bristol.

9.—*A Present-day view of part of the Hotwells.*

to choose Clifton, a place which can be recommended for the magnificent scenery of its immediate neighbourhood; for the rare interest attaching to so much that is left of an ancient city which has taken a large share in the national history; for the ever-varying incidents of a commercial seaport, and the facilities afforded for a variety of charming river and sea trips; for its interesting churches, of which there are a great number; for its many educational advantages; and for the attractions of the surrounding country.

### BIBLIOGRAPHY OF THE HOTWELL WATER.

VENNER (T.). *The baths of Bathe. . . . Whereunto is also annexed a censure concerning the water of Saint Vincents Rocks, neere Bristoll*, 1628; 2nd ed., 1638; 3rd ed., 1650.

JORDEN (E.). *A discourse of naturall bathes and minerall waters, especially of our bathes at Bathe in Sommersetshire*, 1631; 2nd ed., 1632; 3rd ed., 1633.

JOHNSON (T.). *Mercurius Botanicus*, 1634.

FULLER (T.). *The history of the Worthies of England*, 1662.

CLAROMONTIUS (C.). *De aere, locis, et aquis terræ Angliæ*, 1672.

GUIDOTT (T.). *A discourse of Bathe, and the hot waters there. Also some enquiries into the nature of the water of St. Vincent's Rock, near Bristol*, 1676; 2nd ed., 1725.

GUIDOTT (T.). *De thermis Britannicis tractatus*, 1691.

MAPLET (J.). *Cl. viri J. Maplet. . . . epistolarum medicarum specimen de thermarum Bathonensium effectis . . . Edente T. Guidott*, 1694.

UNDERHILL (J.). *Thermologia Bristoliensis*, 1703.

ALLEN (B.). *The natural history of the mineral waters of Great-Britain*, 1711.

ATKYNS (Sir R.). *The ancient and present state of Glocestershire*, 1712; 2nd ed., 1768.

GOLDWIN (W.) *A poetical description of Bristol*, 1712; 2nd and 3rd eds., 1751.

WYNTER (J.). *Cyclus metasyncriticus, or, an essay on chronical diseases, and on the medicinal waters of Bath and Bristol*, 1725; 2nd ed., 1728.

KEIR (P.). *Enquiry into the nature and virtues of the medicinal waters of Bristol, in chronical distempers*, 1739.

SHEBBEARE (J.). *A new analysis of the Bristol waters*, 1740.

RANDOLPH (G.). *An enquiry into the medicinal virtues of Bristol-water*, 1745; 1750.

LINDEN (D. W.). *A treatise on the origin, nature, and virtues, of chalybeat waters and natural hot-baths*, 1748; 2nd ed., 1755; 3rd ed., 1759.

WHITEHEAD (W.). "An hymn to the Nymph of Bristol spring," 1751, (*Plays and Poems*, 1774, vol. ii., pp. 105-25.)

OWEN (E.). *Observations on the earths, rocks, stones and minerals for some miles about Bristol*, 1754.

LUCAS (C.) *An essay on waters*, 1756.

RUTTY (J.). *A methodical synopsis of mineral waters*, 1757.

SUTHERLAND (A.). *The nature and qualities of Bristol-water with reflections on Bath-waters*, 1758; 1788.

RUTTY (J.). *Argument of sulphur or no sulphur in waters discussed, with a comparison of the waters of Aix-la-Chapelle, Bath, and Bristol*, 1762.

SUTHERLAND (A.). *Attempts to revive antient medical doctrines, of waters, of Bath and Bristol waters in particular*, 1763.

SUTHERLAND (A.). *An attempt to ascertain the virtues of Bath and Bristol waters*, 1764.

LUCAS (C.). *Cursory remarks on Sutherland's method of investigating the Bath and Bristol Waters*, [1764].

LINDEN (D. W.). *Reply to Dr. Lucas's remarks on Sutherland's treatise on Bath and Bristol waters*, 1765.

FOTHERGILL (J.). "The use of Bristol water in consumption." *Medical Observations and Inquiries*, 1776, v. 355-9.

JONES (H.). *Clifton: a poem*, 1767, pp. 8-13. (Date printed on title-page, 1667, is an error.); 2nd ed., 1773.

ELLIOT (J.). *An account of the nature and medicinal virtues of the principal mineral waters of Great Britain and Ireland*, 1781; 2nd ed., 1789.

SHIERCLIFF (E.). *Bristol and Hotwell Guide*, 1789.

NOTT (J.). *Of the Hotwell waters, near Bristol*, 1793; 2nd ed. 1797; 3rd ed. [after 1802].

[HEATH (G.)]. *Matthews's New History of Bristol*, 1794.

*An impartial inqury into the nature and qualities of the new saline mineral spa water, at the Tennis court house, Hotwells Road, Bristol.* By a Gentleman of the Faculty. [N.D.: after 1793.]

CARRICK (A.). *Dissertation on the chemical and medical properties of the Bristol Hotwell water*, 1797.

SAUNDERS (W.). *A treatise on the chemical history and medical powers of some of the most celebrated mineral waters*, 1800; 2nd ed., 1805.

MANBY (G. W.). *Fugitive sketches of the history & natural beauties of Clifton, Hotwells and vicinity*, [1802].

NISBET (W.). *A medical guide for the invalid*, 1804.

LATIMER (J.). *The annals of Bristol in the seventeenth, eighteenth, and nineteenth centuries*, 3 vols., 1887-1900.

BEDDOE (J.) "Clifton." *Bristol Medico-Chirurgical Journal*, 1889, vii. 114-22.

NOTES ON THE ILLUSTRATIONS.

8 (facing p. 193). This shows the pump-room built in 1822 and taken down in 1867. It was "sketched from Nature by T. Hulley" and "drawn on stone by H. Jones, R.I.A."

9 (p. 205). This gives a view of a portion of the Hotwells as it appears to-day. It is from a photograph taken by Messrs. Marion and Co. for *Picturesque Bristol and Clifton*, published by the Scholastic Trading Co., Bristol, and to their kindness I am indebted for permission to use it. The view shows a portion of the Colonnade which was erected about 1760. (See illustration on p. 23.) The bridge was finished in 1864. Its height from low water is 280 feet, and the span is 702 feet.

---

Mr. F. W. Stoddart, the Public Analyst of Bristol, has very kindly supplied me with these Notes on some of the Analyses of the Hotwell Water which are referred to in the foregoing paper:—

The earliest account by Venner notes the presence of sulphur (*i.e.* sulphuretted hydrogen) and nitre, and further that it was likely to lose the former on keeping.

Guidott's analysis excluded volatile matters; but that the water to his knowledge contained sulphuretted hydrogen appears from his opinion that "a nitro-sulphureous salt" was present. The mode of identification of the carbonate of lime by igniting the insoluble residue to the oxide, slaking and combining with sulphur, and finally decomposing the latter with an acid, is ingenious and correct. The method of analysis at this date consisted practically of separation by solvents, just as the proximate analysis of organic substance does at the present time.

It is noticeable that from Guidott's time, and after the erection of a pump-room, no mention is made of "sulphur." Was the true spring lost at this time? Allen's account is very obscure and hardly recognisable as referring to this water, which has by every other authority been found to contain much carbonate of lime and other salts, if not altogether deserving of Strother's grandiose description.

Sutherland's analysis is also much opposed to ancient and modern investigation. It is difficult to understand what is meant by "volatile vitriolic acid." Sulphur dioxide would seem to fit in best with the description, but would probably have been identified more distinctly.

Carrick's analysis, the first in modern form, represents the water as being of much the same character as at present, though there is more combined sulphuric acid.

Saunders's remarks, contradictory as they are, are an illustration of the curious fact that certain hard waters do undoubtedly get the reputation of being soft. In modern times the water has certainly maintained with great uniformity the composition given by Dr. Herapath, and has probably remained constant in this respect since the end of the seventeenth century.

## Figures and Tables

**8 f1:**
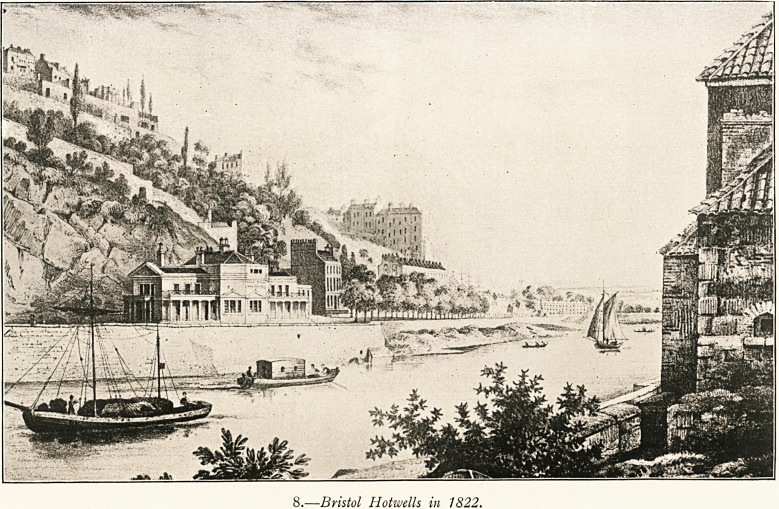


**9 f2:**